# Studying Vertical Microbiome Transmission from Mothers to Infants by Strain-Level Metagenomic Profiling

**DOI:** 10.1128/mSystems.00164-16

**Published:** 2017-01-17

**Authors:** Francesco Asnicar, Serena Manara, Moreno Zolfo, Duy Tin Truong, Matthias Scholz, Federica Armanini, Pamela Ferretti, Valentina Gorfer, Anna Pedrotti, Adrian Tett, Nicola Segata

**Affiliations:** a Centre for Integrative Biology, University of Trento, Trento, Italy; b Azienda Provinciale per i Servizi Sanitari, Trento, Italy; Argonne National Laboratory

**Keywords:** infant microbiome, metagenomics, microbial ecology, microbial genomics, vertical microbiome transmission

## Abstract

The gut microbiome becomes shaped in the first days of life and continues to increase its diversity during the first months. Links between the configuration of the infant gut microbiome and infant health are being shown, but a comprehensive strain-level assessment of microbes vertically transmitted from mother to infant is still missing. We collected fecal and breast milk samples from multiple mother-infant pairs during the first year of life and applied shotgun metagenomic sequencing followed by computational strain-level profiling. We observed that several specific strains, including those of *Bifidobacterium bifidum*, *Coprococcus comes*, and *Ruminococcus bromii*, were present in samples from the same mother-infant pair, while being clearly distinct from those carried by other pairs, which is indicative of vertical transmission. We further applied metatranscriptomics to study the *in vivo* gene expression of vertically transmitted microbes and found that transmitted strains of *Bacteroides* and *Bifidobacterium* species were transcriptionally active in the guts of both adult and infant. By combining longitudinal microbiome sampling and newly developed computational tools for strain-level microbiome analysis, we demonstrated that it is possible to track the vertical transmission of microbial strains from mother to infants and to characterize their transcriptional activity. Our work provides the foundation for larger-scale surveys to identify the routes of vertical microbial transmission and its influence on postinfancy microbiome development.

**IMPORTANCE** Early infant exposure is important in the acquisition and ultimate development of a healthy infant microbiome. There is increasing support for the idea that the maternal microbial reservoir is a key route of microbial transmission, and yet much is inferred from the observation of shared species in mother and infant. The presence of common species, *per se*, does not necessarily equate to vertical transmission, as species exhibit considerable strain heterogeneity. It is therefore imperative to assess whether shared microbes belong to the same genetic variant (i.e., strain) to support the hypothesis of vertical transmission. Here we demonstrate the potential of shotgun metagenomics and strain-level profiling to identify vertical transmission events. Combining these data with metatranscriptomics, we show that it is possible not only to identify and track the fate of microbes in the early infant microbiome but also to investigate the actively transcribing members of the community. These approaches will ultimately provide important insights into the acquisition, development, and community dynamics of the infant microbiome.

## Abstract (precis)

Early infant exposure is important in the acquisition and ultimate development of a healthy infant microbiome. There is increasing support for the idea that the maternal microbial reservoir is a key route of microbial transmission, and yet much is inferred from the observation of shared species in mother and infant. The presence of common species, *per se*, does not necessarily equate to vertical transmission, as species exhibit considerable strain heterogeneity. It is therefore imperative to assess whether shared microbes belong to the same genetic variant (i.e., strain) to support the hypothesis of vertical transmission. Here we demonstrate the potential of shotgun metagenomics and strain-level profiling to identify vertical transmission events. Combining these data with metatranscriptomics, we show that it is possible not only to identify and track the fate of microbes in the early infant microbiome but also to investigate the actively transcribing members of the community. These approaches will ultimately provide important insights into the acquisition, development, and community dynamics of the infant microbiome.

## INTRODUCTION

The community of microorganisms that dwell in the human gut has been shown to play an integral role in human health (1–4), facilitating, for instance, the harvesting of nutrients that would otherwise be inaccessible (5), modulating the host metabolism and immune system (6), and preventing infections by occupying the ecological niches that could otherwise be exploited by pathogens (7). The essential role of the intestinal microbiome is probably best exemplified by the successful treatment of dysbiotic states, such as chronic life-threatening *Clostridium difficile* infections, using microbiome transplantation therapies (8–10).

The gut microbiome is a dynamic community shaped by multiple factors throughout an individual’s life, possibly including prebirth microbial exposure. The early development of the infant microbiome has been proposed to be particularly crucial for longer-term health (11–13), and a few studies have investigated the factors that are important in defining its early structure (14–17). In particular, gestational age at birth (17), mode of delivery (14, 15), and early antibiotic treatments (18) have all been shown to influence the gut microbial composition in the short term and the pace of its development in the longer term.

Vertical transmission of bacteria from the body and breast milk of the mother to her infant has gained attention as an important source of microbial colonization (14, 19–21) in addition to the microbial organisms obtained from the wider environment (22, 23), including the delivery room (24). Results from early cultivation-based and cultivation-free methods (16S rRNA community profiling and a single metagenomic study) have indeed suggested that the mother could transfer microbes to the infant by breastfeeding (25) and that a vaginal delivery has the potential of seeding the infant gut with members of the mother’s vaginal community (11, 14, 26, 27) that would not be available via caesarean section. However, a more in-depth analysis is required to elucidate the role of vertical transmission in the acquisition and development of the infant gut microbiome.

Current knowledge of the vertical transmission of microbes from mothers to infants has hitherto focused on the cultivable fraction of the community (28) or lacked strain-level resolution (11). Many microbial species are common among unrelated individuals (29); therefore, in instances where a species is identified in both mother and infant (13, 30), it remains inconclusive if this is due to vertical transmission. Strain-level analysis has shown that different individuals are associated with different strains of common species (31, 32), and it is therefore crucial to profile microbes at the strain level to ascertain the most probable route of transfer. This has been performed only for specific microbes by cultivation methods (16, 28), but many vertically transmitted microorganisms remain hard to cultivate (16); thus, the true extent of microbial transmission remains unknown. A further crucial aspect, still largely unexplored, is the fate of vertically acquired strains: if they are transcriptionally active rather than merely transient, that may suggest possible colonization of the infant intestine. Although studies have described the transcriptional activity of intestinal microbes under different conditions (33–36), no studies have applied metatranscriptomics to characterize the activity of vertically transmitted microbes *in vivo*.

In this work, we present and validate a shotgun metagenomic pipeline to track mother-to-infant vertical transmission of microbes by applying strain-level profiling to members of the mother and infant microbiomes. Moreover, we assessed the transcriptional activity of vertically transmitted microbes to elucidate if transferred strains are not only present but also transcriptionally active in the infant gut.

## RESULTS AND DISCUSSION

We analyzed the vertical transmission of microbes from mother to infant by enrolling 5 mother-infant pairs and collecting fecal samples and breast milk (see Materials and Methods) when each infant was 3 months of age (time point 1). Two mother-infant pairs (pair 4 and pair 5) were additionally sampled at 10 months postbirth (time point 2), and one pair (pair 5) was sampled at 16 months postbirth (time point 3; see Fig. S1 in the supplemental material). We applied shotgun metagenomic sequencing to all 24 microbiome samples (8 mother fecal samples, 8 infant fecal samples, and 8 milk samples), generating 1.2 G reads (average, 39.6 M reads/sample; standard deviation [SD], 28.7 M reads/sample) (see Table S1 in the supplemental material). Metatranscriptomics (average, 90.55 M reads/sample; SD, 46.86 M reads/sample) was also applied on fecal samples of two pairs (pair 4 at time point 2 and pair 5 at time point 3) to investigate the differential expression profiles of the bacterial strains in the gut of mothers and their infants.

10.1128/mSystems.00164-16.1 FIG S1  Study design. A schematic representation of the mother-infant pairs involved in the study, the sample types, and the time points considered is presented. Marked with the “RNA” label, the mother-infant pairs for which stool metatranscriptomes were produced are indicated. Download FIG S1, EPS file, 0.5 MB. Copyright © 2017 Asnicar et al. 2017 Asnicar et al. This content is distributed under the terms of the Creative Commons Attribution 4.0 International license.

10.1128/mSystems.00164-16.2 TABLE S1  Sample metadata and raw data. The table reports the sample metadata, the efficiency of extraction, and information about the raw reads. Download TABLE S1, XLSX file, 0.01 MB. Copyright © 2017 Asnicar et al. 2017 Asnicar et al. This content is distributed under the terms of the Creative Commons Attribution 4.0 International license.

10.1128/mSystems.00164-16.7 TABLE S2  MetaPhlAn2 abundance profiles. The table reports relative abundances of different microbes in metagenomic samples, as profiled with MetaPhlAn2. Download TABLE S2, XLSX file, 0.1 MB. Copyright © 2017 Asnicar et al. 2017 Asnicar et al. This content is distributed under the terms of the Creative Commons Attribution 4.0 International license.

### Shared mother-infant microbial species.

**FIG 1:**
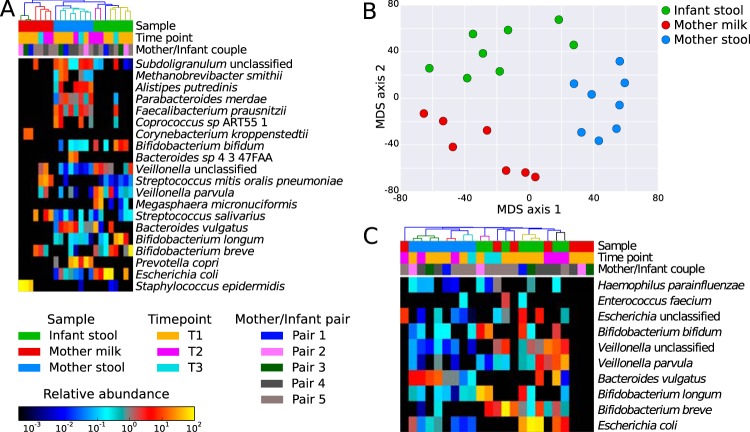
Microbial composition of mother and infant samples and shared bacteria within mother-infant pairs. (A) Quantitative microbial taxonomic composition of the metagenomic samples from milk and fecal samples of mothers and infants as estimated by MetaPhlAn2 analysis (77) (only the 20 most abundant species are indicated). Milk samples present low microbial richness compared to fecal samples. (B) Ordination plot of microbiome composition showing clustering of the three different sample types: mother feces, infant feces, and breast milk samples. The two infant samples close to the cluster of mother feces and in between the clusters of mothers and infants are from later time points, denoting the convergence of the infant microbiome toward an adult-like one. (C) The abundances of the 10 microbial species detected (>0.1% abundance) in at least one infant and the respective mother (shared species have been identified on the basis of samples from time point 1 [T1] only).

In our cohort, the infant intestinal microbiome was dominated by *Escherichia coli* and *Bifidobacterium* spp., such as *B. longum*, *B. breve*, and *B. bifidum* (Fig. 1A and S2). These species in some cases reached abundances higher than 75% (e.g., *E. coli* at 85.2% in infant pair 3 at time point 1 and *B. breve* at 78.8% in infant pair 5 at time point 1), which is consistent with previous observations (12, 37, 38). As expected, the intestines of the mothers had a greater microbial diversity than those of the infants, with high abundances of *Prevotella copri*, *Clostridiales* (e.g., *Coprococcus* spp. and *Faecalibacterium prausnitzii*), and *Bacteroidales* (e.g., *Parabacteroides merdae* and *Alistipes putredinis*). Interestingly, the postweaning microbiome of infant of pair 5 (time point 3, 16 months postbirth) had already shifted toward a more “mother-like” composition (Fig. 1B), with an increase in diversity and the appearance of *Parabacteroides merdae*, *Coprococcus* spp., and *Faecalibacterium prausnitzii* (13, 38). Nevertheless, this 16-month-old infant still retained some infant microbiome signatures, such as a high abundance of bifidobacteria that were present at only low levels in the mothers’ samples (Fig. 1A and C).

10.1128/mSystems.00164-16.3 FIG S2  Extensive taxonomic profiling of the top 100 species from MetaPhlAn2 analysis and the five most highly represented niche-specific species. (A) The heat map shows differences in terms of species richness between mother, infant, and milk metagenomes. In particular, the milk samples have very low microbial diversity, especially at time point 1. The microbiomes of the mothers have instead higher diversity than both the milk microbiomes and the infant microbiomes. (B) We selected the five most highly represented species on average for each sample type (mother milk, mother stool, and infant stool) and plotted their average abundances in each niche. Each sample type is dominated by its five most highly represented species that are, in general, underrepresented in the other niches. Download FIG S2, EPS file, 1.8 MB. Copyright © 2017 Asnicar et al. 2017 Asnicar et al. This content is distributed under the terms of the Creative Commons Attribution 4.0 International license.

We extracted and successfully sequenced microbial DNA from 7 of 8 milk samples. Microbial profiling of milk samples was hindered by a high abundance of interfering molecules (proteins, fats, proteases—e.g., plasmin—and calcium ions) (39–41) that affected the efficiency of the extraction and amplification steps. Even so, we obtained an average of 3.08 Gb (SD, 1.5 Gb) per sample, of which 26 Mb (SD, 56 Mb) were from nonhuman reads (a level higher than that seen in the only other metagenomic study) (42) (see Table S1).

Milk samples had limited microbial diversity at the first sampling time (time point 1, 3 months postbirth) and included skin-associated bacteria such as *Corynebacterium kroppenstedtii* and *Staphylococcus epidermidis*. Cutaneous taxa, however, were observed in only low abundances in the gut microbiome of infants, confirming that skin microbes are not colonizers of the human gut (Fig. 1A). At later time points, the milk samples were enriched in *B. breve* and in bacteria usually found in the oral cavity, such as *Streptococcus* and *Veillonella* spp. The presence of oral taxa in milk has been previously observed by 16S rRNA sequencing (14, 20, 21, 25) and shotgun metagenomics (42). This could be caused by retrograde flux into the mammary gland during breastfeeding (43) whereby cutaneous microbes of the breast and from the infant oral cavity are transmitted to the breast glands (44). However, this remains a hypothesis because no oral samples were collected in this study. These observations are summarized in the ordination analysis (Fig. 1B), in which the different samples (infant feces, mother feces, and milk) clustered by type, with weaning representing a key factor in the shift from an infant to an adult-like microbiome structure (13, 38, 45).

Comparing the species present in both the mother and infant pairs (Fig. 1C), we observed that many shared species (e.g., *Escherichia*, *Bifidobacterium*, and *Veillonella* spp.) occurred at a much higher abundance in the infant than in the mother, possibly due to the lower level of species diversity and therefore to competition in the gut. *Bacteroides vulgatus* was found at relatively high abundance (average, 16.3%; SD, 13%) in both the infant and the mother of pair 4 at both time point 1 and time point 2. The presence of shared species in mother-infant pairs observed here and elsewhere (14, 16, 17, 25, 46) confirms that mothers are a potential reservoir of microbes vertically transmissible to infants, but it remains unproven whether the same strain is transmitted to the infant from the mother or if an alternative transmission route is involved.

### Strains shared between mothers and infants are indicative of vertical transmission.

While different individuals have a core of shared microbial species, it has been shown that these common species consist of distinct strains (31, 32). To analyze microbial transmission, it is therefore crucial to assess whether a mother and her infant harbor the same strain. To this end, we further analyzed the metagenomic samples at a finer strain-level resolution. This was achieved by applying a recent strain-specific pangenome-based method called PanPhlAn (31), as well as a genetics-based method called StrainPhlAn (D. T. Truong, A. Tett, E. Pasolli, C. Huttenhower, and N. Segata, submitted for publication) (see Materials and Methods), which identifies single-nucleotide variants (SNVs) in species-specific marker genes.

**FIG 2:**
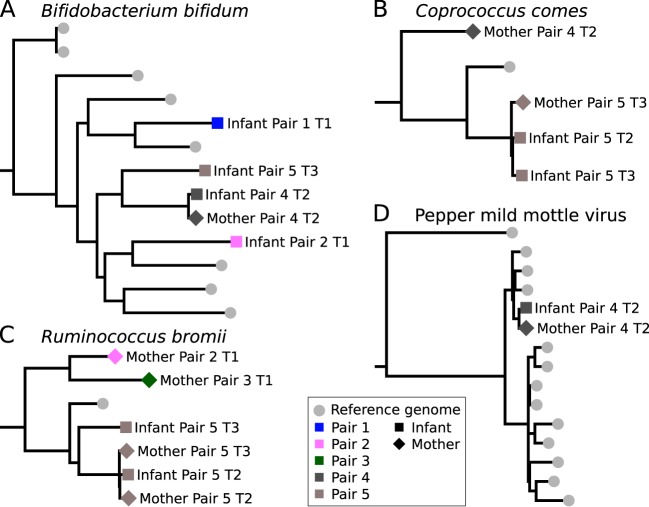
Strain-level phylogenetic trees for microbes present in both the mother and infant. Phylogenetic trees were built by the StrainPhlAn method using species-specific markers confirming the presence of the same strain in the mother and infant intestinal microbiomes, thus suggesting vertical transmission. Available reference genomes were included in the phylogenetic trees. Here we report three bacterial species, namely, (A) *Bifidobacterium bifidum*, (B) *Coprococcus comes*, and (C) *Ruminococcus bromii*, and the most abundant viral species found in pair 4, (D) pepper mild mottle virus. Other species-specific phylogenetic trees (*B. adolescentis*, *B. breve*, and *B. longum*) are reported in Fig. S3.

Using the SNV-based analysis, we observed considerable strain-level heterogeneity in the species present in the intestines of the mothers also with respect to available reference genomes (Fig. 2; see also Fig. S3 in the supplemental material). This heterogeneity was not observed within the mother-infant pairings, as in the case of *Bifidobacterium* spp., *Ruminococcus bromii*, and *Coprococcus comes*. The infant of pair 4 at time point 2, for example, harbored a strain of *B. bifidum* that matched his mother’s at 99.96% sequence identity and yet was clearly distinct from the *B. bifidum* strains of other infants in the cohort (Fig. 2A), which differed by at least 0.6% of the nucleotides. The observation that the *B. bifidum* strains from the mother and the infant of pair 4 were too similar to be consistent with the observed strain-level variation across subjects in the cohorts was highly statistically significant (*P* value, 4.7e−40) (see Fig. S4). This was also true for the *C. comes* (*P* value, 1.9e−3) (99.87% intrapair similarity and 1.6% and 1.61% divergence compared to the closest strain and the average value, respectively) (Fig. 2B) and *R. bromii* (*P* value, 4.9e−8) (99.93% similarity and 1.53% and 2.63% diversity—same as described above) (Fig. 2C) strains that were shared by pair 5. Mother-infant sharing of the same strain was also confirmed by strain-level pangenome analysis (31) that showed that the strains from the same pair carried the same unique gene repertoire (see Fig. S5). It is accepted that, while the possibility of independent acquisition of strains from a shared environmental source cannot be excluded, the finding that mother-infant pairs have shared strains represents strong evidence of vertical microbiome transmission. On average, we could reconstruct and observe vertical transmission from mother to infant for 14% of the species found to be shared within mother and infant pairings.

10.1128/mSystems.00164-16.4 FIG S3  Strain-level analysis showing vertical transmission from mother to infant of bifidobacterium species. The phylogenetic trees were produced by applying StrainPhlAn for the following species: (A) *Bifidobacterium adolescentis*, (B) *Bifidobacterium breve*, and (C) *Bifidobacterium longum*. In each tree, a clade containing one (or more) samples of the mother and infant of the same pair is observed. This suggests that the strain is shared between mother and infant, hence suggesting vertical transmission. Download FIG S3, EPS file, 0.1 MB. Copyright © 2017 Asnicar et al. 2017 Asnicar et al. This content is distributed under the terms of the Creative Commons Attribution 4.0 International license.

10.1128/mSystems.00164-16.5 FIG S4  Distribution of SNV rates of *Bifidobacterium bifidum*. We computed the SNV rates of the strains of *B. bifidum* reconstructed with StrainPhlAn (the phylogenetic tree is presented in Fig. 2A). The two strains of the mother and the infant of pair 4 at time point 2 have an SNV rate of 0.04. The first bin has a frequency of two because it comprises not only the SNV rate of pair 4 at time point 2 but also the SNV rate of the two reference genomes reported in the upper part of the phylogenetic tree in Fig. 2A. The two reference genomes have an SNV rate of 0, meaning that they are identical. Download FIG S4, EPS file, 0.2 MB. Copyright © 2017 Asnicar et al. 2017 Asnicar et al. This content is distributed under the terms of the Creative Commons Attribution 4.0 International license.

10.1128/mSystems.00164-16.6 FIG S5  Strain-level analysis by applying PanPhlAn confirms vertical transmission. We applied PanPhlAn to validate the results obtained with StrainPhlAn (Fig. 2 and S3). The pangenome-based strain-level analysis shows the presence and absence (in red and yellow, respectively) of the species-specific gene families of the following species: *B. bifidum*, *C. comes*, *R. bromii*, *B. adolescentis*, *B. breve*, and *B. longum*. Samples are clustered according to hierarchical clustering based on the Euclidian distance of the samples’ pangenome profiles. Download FIG S5, EPS file, 0.1 MB. Copyright © 2017 Asnicar et al. 2017 Asnicar et al. This content is distributed under the terms of the Creative Commons Attribution 4.0 International license.

Strain transmission does not, however, exclude later replacement of the vertically acquired organisms, as we highlighted by looking at the postweaning time point in our cohort (pair 5 at time point 3) which harbored the highest number of shared species, with 70.4% present in the infant and mother (at a relative abundance of >0.1%, according to the MetaPhlAn2 profiles). A proportion (11%) of these common species were shown to be the same strain (Fig. 2; see also Fig. S3 in the supplemental material), according to both PanPhlAn and StrainPhlAn analyses (see, for example, the data from *B. adolescentis* and *C. comes*) (Fig. 2; see also Fig. S3 and S5). However, some strains that were shared at earlier time points were replaced at time point 3. Of note, the *R. bromii* strain found in an infant at time point 3 was different from that found at time point 2, and both strains were distinct from the strain observed in the mother at both time points (Fig. 2C). This was also observed for the latter infant time point for *B. breve* (see Fig. S3B) and *B. longum* (see Fig. S3C). Although it is not possible to generalize these results because of the small sample size, these replacement events suggest that originally acquired maternal strains can subsequently be replaced (47, 48).

We then extended our analysis to the viral organisms detectable from metagenomes and metatranscriptomes, as viruses have the potential to be vertically transmitted also. The DNA viruses identified from our metagenome samples largely consisted of bacteriophages of the *Caudovirales* order, a common order of tailed bacteriophages found in the intestine (3, 49). We identified *Enterobacter* and *Shigella* phages as the most prevalent phages among the tested samples, in agreement with the high prevalence of members of the *Enterobacteriaceae* family and particularly of members of the *Escherichia* genus (see Fig. 1A and Table S3). We also identified crAssphage at high breadth of coverage (50) and provided further evidence for the hypothesis that the *Bacteroides* genus is the host for this virus (50), as the microbiome of crAssphage-positive mothers was enriched in *B. vulgatus* (see Fig. 1A and Table S3). However, the low breadth of coverage for many of the DNA viruses made it difficult to identify pair-specific phage variants (see Table S3). Analysis of the RNA viruses from the metatranscriptomic samples identified instead the presence of an abundant pepper mild mottle virus (PMMoV), a single-stranded positive-sense RNA virus of the genus *Tobamovirus*, in all of the four metatranscriptomes from pairs 4 and 5. Surprisingly, transcripts from the PMMoV were found in greater abundance than all the other microbial transcripts found for the mother of pair 4. PMMoV has already been reported in the gut microbiome (51–53), and other related viruses of the same family have been shown to be able to enter and persist in eukaryotic cells (54, 55). The high abundance of PMMoV in mother-infant pair 4 allowed us to reconstruct its full genome (99.9%) and to perform a phylogenetic analysis demonstrating that the mother and the infant shared identical PMMoV strains, which were clearly distinct from the PMMoV reference genomes (27 SNVs in total; Fig. 2D). Although the coverage was lower, the same evidence of a shared PMMoV strain was observed within pair 5. The analysis of PMMoV polymorphisms within each sample also suggests the coexistence of different PMMoV haplotypes in the same host (Fig. S6). Although vertical transmission of RNA viruses and PMMoV specifically would be intriguing, because of the age and dietary habits of the infants (see Table S1) this finding could be related to the exposure to a common food source (56). Our analysis of the virome characterized directly from shotgun metagenomics thus highlighted that viruses can be tracked across mother-infant microbiomes also and that experimental virome enrichment protocols (57, 58) have the potential to provide an even clearer snapshot of viral vertical transmission.

10.1128/mSystems.00164-16.8 FIG S6  Read alignment of pepper mild mottle virus (PMMoV) for both pair 4 and pair 5. Alignments of mother and infant of both pair 4 and pair 5 against the PMMoV reference genome are presented, showing variations highlighted in red (mother) and blue (infant) for a window of 160 bp. Pair 4 data (from position 3216 to position 3376 in the PMMoV genome) show the agreement between the mother and infant variations, suggesting that they share the same strain of the PMMoV. Pair 5 data (from position 4450 to position 4610 in the PMMoV genome) show the presence of more than one viral strain in the mother. Variations in the infant data are coherent with data from the mother, with the former harboring only a subset of the mother’s strains. Download FIG S6, EPS file, 0.1 MB. Copyright © 2017 Asnicar et al. 2017 Asnicar et al. This content is distributed under the terms of the Creative Commons Attribution 4.0 International license.

10.1128/mSystems.00164-16.10 TABLE S3  DNA virus abundance data. The table shows the breadth of coverage and the average depth of coverage for the DNA viruses found in the metagenomes. Download TABLE S3, XLSX file, 0.01 MB. Copyright © 2017 Asnicar et al. 2017 Asnicar et al. This content is distributed under the terms of the Creative Commons Attribution 4.0 International license.

### Differences in the overall levels of functional potential and expression in mothers and infants.

**FIG 3:**
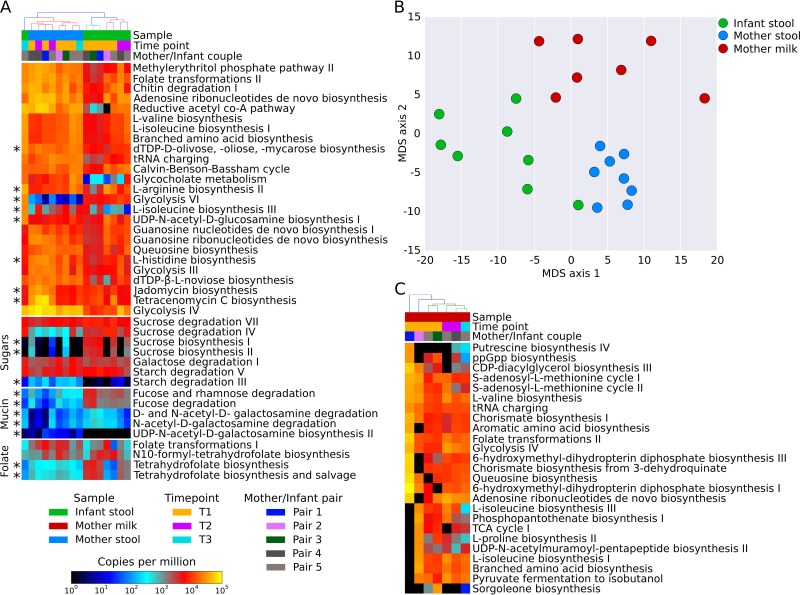
Functional potential analyses. (A) HUMAnN2 heat map reporting the 25 most abundant pathways in the fecal samples of mothers and infants. Specific pathways of interest (sugars, mucin, and folate metabolism) are added at the bottom. The asterisk (*) near the heat map highlights statistically significant pathways. (B) Multidimensional scaling (MDS) result from functional potential profiles, showing the differences between fecal samples of mothers and infants and milk samples. In particular, the infant feces point in the mother feces cluster corresponds to time point 3 of pair 5, showing a shift from the infant microbiome toward an adult-like microbiome. (C) HUMAnN2 results for the 25 most abundant pathways found only in the milk samples. TCA, tricarboxylic acid.

The physiology of the mammary gland (milk) as well as the adult and infant intestine is reflected by niche-specific microbial communities as reported above and in previous studies (11, 13, 15, 20, 21, 44, 45). To characterize the overall functional potential of the microbial communities inhabiting these niches, we complemented the taxonomic analysis above by employing HUMAnN2 (see Materials and Methods). As expected, there was considerable overlap in the functionality of the gut microbiomes of the mothers and infants (Fig. 3A), with 87% of pathways present in mother and infant, 50% of which were significantly different in abundance (at an alpha value of 0.05). Nevertheless, there were notable differences. For instance, the microbiomes of the infants showed a higher potential for utilization of intestinal mucin as a carbon source (*P* value, 0.016) and for folate biosynthesis (*P* value, 1.8e−6) while displaying a lower potential for starch degradation (*P* value, 9.8e−6), consistent with previous observations (12, 59–62). Mucin utilization, specifically by infant gut microbial communities, is reflective of the higher abundance of mucin-degrading bifidobacteria observed from the taxonomic analyses described above (12, 59–61), whereas increased folate biosynthesis (12, 59, 60, 62) and decreased starch degradation (5) have been purported to represent responses to the limited dietary intake in infants compared to adults. Interestingly, the intestinal samples from the postweaning infant of pair 5 (16 months postbirth) clustered together with the adults’ intestinal samples (Fig. 3B), suggesting that the shift toward an adult-like microbiome observed in the taxonomic profiling (Fig. 1B) is also reflected by or is a consequence of a change in community functioning. Among the most prevalent pathways in the milk microbiomes that we observed were those involved in galactose and lactose degradation (63), as well as in biosynthesis of aromatic compounds (Fig. S7A). This was specifically true for production of chorismate, a key intermediate for the biosynthesis of essential amino acids and vitamins found in milk (62) (Fig. 3C and S7A).

10.1128/mSystems.00164-16.9 FIG S7  Functional potential biomarker analysis and metabolic pathway expression in mother and infant of pair 5 at time point 3. (A) Degradation and biosynthesis pathways revealed by HUMAnN2 results processed with LEfSe to investigate differentially expressed pathways and functions. Biomarkers for the three classes are reported in different colors as follows: green, infant feces; red, mother milk; blue, mother feces. The sizes of the clades represent the linear discriminant analysis (LDA) effect sizes assigned by LEfSe (see Materials and Methods). Infants were harboring mainly sugar degraders and showed a higher potential for degradation of aromatic compounds and biosynthesis of cofactors. The microbial communities from the mothers showed instead higher representation of pathways involved in the biosynthesis of carbohydrates and antibiotics and in the degradation of C1 compounds and amino acids. (B and C) Metatranscriptomic analysis of samples from the mother and infant of pair 5 at time point 3 performed with both HUMAnN2 and PanPhlAn. (B) Scatterplots showing the transcription rates of metabolic pathways of different species and genera of interest obtained from HUMAnN2. (C) Comparison between transcription rates of gene families from PanPhlAn data. Download FIG S7, PDF file, 2.5 MB. Copyright © 2017 Asnicar et al. 2017 Asnicar et al. This content is distributed under the terms of the Creative Commons Attribution 4.0 International license.

**FIG 4:**
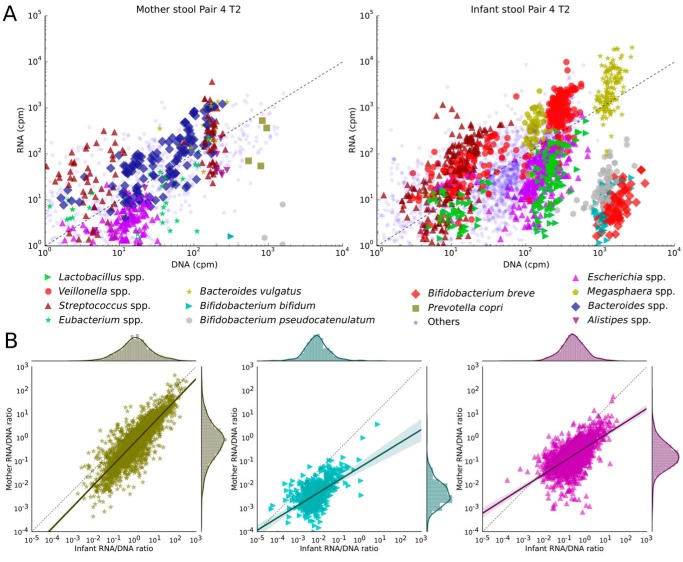
Transcription levels of metabolic pathways and genes in mother and infant pair 4 at time point 2. (A) Scatterplots showing the transcription rates of metabolic pathways of shared and nonshared species and genera of interest for both the mother and infant of pair 4 at time point 2. (B) Comparison between transcription rates of gene families in mother and infant gut microbiomes.

To further evaluate the functional capacity of the gut-associated microbiomes and analyze the *in vivo* transcription, we performed metatranscriptomics analyses of the feces of two mother-infant pairs (see Materials and Methods). HUMAnN2 was used to identify differences in the transcriptional levels of pathways in the gut of the mothers and infants. The most notable global difference was that fermentation pathways were highly transcribed in the mother compared to that of the infant. This reflects the transition of the gut from an aerobic to an anaerobic state and the associated shift from facultative anaerobes to obligate anaerobes over the first few months of life (64, 65). The same is true for pathways involved in starch degradation, which were not only poorly represented in the metagenomes but also negligibly expressed in the infants’ transcriptomes. What is evident is that the transcriptional patterns for different members differed considerably, as illustrated for pair 4 and pair 5 (Fig. 4A and S7B, respectively). For example, we observed in the infant of pair 4 that *B. vulgatus* was more transcriptionally active (average of 2.7 [SD, 2.5] normalized transcript abundance [NTA]; see Materials and Methods) than both *E. coli* (245-fold change [average, 0.4 SD and 0.6 NTA]) and *Bifidobacterium* spp. (6.6-fold change [average, 0.01 SD and 0.01 NTA]). Although these differences were statistically significant (*P* values were lower than 1e−50 in both cases), their physiological significance remains unclear.

### Strain-specific transcriptional differences in mothers and infants.

To further explore the transcriptional activity of the intestinal microbiomes and, more specifically, to ascertain which individual microbial members are transcriptionally active in the gut, we employed the strain-specific metatranscriptomic approach implemented in PanPhlAn (31) (see Materials and Methods). Of particular interest is the transcriptional activity of the shared mother-infant strains that, based on our strain-level analyses, are likely to have been vertically acquired by the infant by the maternal route. Such transcriptional analyses can clarify whether these transmitted strains were not only present in the infant gut but also functioning, therefore suggesting that the transmitted strains could have potentially colonized. For three transmitted species in pair 4 (*B. vulgatus*, *E. coli*, and *B. bifidum*), we show that they were active in both the mother intestine and the infant intestine (Fig. 4B). Of note is that *B. bifidum* was more active in the infant than in the mother (2.5-fold change; Fig. 4B), which was expected as this species is a known early colonizer of the infant gut (12, 37, 38). Interestingly, the *B. bifidum* strain of pair 5 showed the opposite behavior (Fig. S7C). We postulate that this was because the infant of pair 5 was of postweaning age (10% breast milk diet) compared to the infant of pair 4 (90% breast milk diet) and that the difference reflects the change in substrate availability from breast milk to solid food, which might have a detrimental effect on the bifidobacterial population (38, 66, 67). Moreover, in support of our metagenomics analyses indicating that the microbiome of infant of pair 5 was shifting toward a more adult-like structure (Fig. 1B), we observed high transcriptional activity for *R. bromii*, a species commonly associated with adults, which could be seen as a hallmark of this transition (68, 69).

It is well established that metatranscriptomic profiling provides a more accurate account of the actual community functioning than metagenomics alone. Here we show that the combination of the two approaches affords the exploration of which members not only are transmitted but also are actively participating in the community and therefore offers a more detailed account of the microbial community dynamics.

### Conclusions.

Human-associated microbiomes are complex and dynamic communities that are continuously interacting with the host and are under the influence of environmental sources of microbial diversity. Identifying and understanding the transmission from these external sources are crucial to understanding how the infant gut is colonized and ultimately develops an adult-like composition. However, detecting direct transmission is not a trivial task: many species are ubiquitous in host-associated environments and in the wider environment alike, and yet they comprise a myriad of different strains and phenotypic capabilities. Therefore, detection of microbial transmission events requires the ability to characterize microbes at the strain level. The epidemiological tracking of pathogens by cultivation-based isolate sequencing has proven successful (70, 71), but it relies on time-consuming protocols and can focus on only a limited number of species. In contrast, while there have been some examples of strain-level tracking from metagenomic data (71, 72), this remains challenging. In this study, we developed methods for identifying the vertical flow of microorganisms from mothers to their infants and showed that mothers are sources of microbes that might be important in the development of the infant gut microbiome.

We demonstrated that high-resolution computational methods applied to shotgun metagenomic and metatranscriptomic data enable the tracking of strains and strain-specific transcriptional patterns across mother-infant pairs. In our cohort of five mother-infant pairs, we detected several species with substantial genetic diversity between different pairs but identical genetic profiles in the mother and her infant, indicative of vertical transmission. These include some bifidobacteria typical of the infant gut (i.e., *B. longum*, *B. breve*, *B. bifidum*, and *B. adolescentis*) but also *Clostridiales* species usually found in the adult intestine (i.e., *R. bromii* and *C. comes*) and viral organisms. These results confirm that the infant receives a maternal microbial imprinting that might play an important role in the development of the gut microbiome in the first years of life.

The strain-level investigation of vertically transmitted microbes was followed by characterization of the transcriptional activity of the transmitted strains in the mother and infant environments. We found that the transcriptional patterns of strains shared within the single pairs were different between mother and infant, suggesting successful adaptation of maternally transmitted microbes to the infant gut.

Taking the results together, our work provides preliminary results and methodology to expand our knowledge of how microbial strains are transmitted across microbiomes. Expanding the cohort size and considering other potential microbial sources of transmission, such as additional mother and infant body sites, as well as other family members (i.e., fathers and siblings) and environments (hospital and house surfaces), will likely shed light on the key determinants in early infant exposure and the seeding and development of the infant gut microbiome.

## MATERIALS AND METHODS

### Sample collection and storage.

In total, five mother-infant pairs were enrolled. Fecal samples and breast milk were collected for all pairs at 3 months (time point 1); additional samples were collected for pair 4 and pair 5 at 10 months (time point 2) and for pair 5 only at 16 months (time point 3) (see Table S1 and Fig. S1 in the supplemental material). All aspects of recruitment and sample and data processing were approved by the local ethics committee. Fecal samples were collected from mothers and infants in sterile feces tubes (Sarstedt, Nümbrecht, Germany) and immediately stored at −20°C. In those cases where metatranscriptomics was applied, a fecal aliquot was removed prior to freezing the remaining feces. This aliquot was stored at 4°C, and the RNA was extracted within 2 h of sampling to preserve RNA integrity. Milk was expressed and collected midflow by mothers into 15-ml centrifuge tubes (VWR, Milan, Italy) and immediately stored at −20°C. Within 48 h of collection, all milk samples and feces samples were moved to storage at −80°C until processed.

### Extraction of nucleic acids for metagenomic analysis.

DNA was extracted from feces using a QIAamp DNA stool minikit (Qiagen, Netherlands). Milk DNA was extracted using a PowerFood microbial DNA isolation kit (Mo Bio, Inc., CA). Both procedures were performed according to the specifications of the manufacturers. Extracted DNA was purified using an Agencourt AMPure XP kit (Beckman Coulter, Inc., CA). Metagenomic libraries were constructed using a Nextera XT DNA library preparation kit (Illumina, CA, USA) according to manufacturer instructions and were sequenced on a HiSeq 2500 platform (Illumina, CA, USA) at an expected sequencing depth of 6 Gb/library.

### Extraction of nucleic acids for metatranscriptomic analysis.

Fecal samples for metatranscriptomic profiling were pretreated as described previously (73). Briefly, 110 μl of lysis buffer (30 mM Tris·Cl, 1 mM EDTA [pH 8.0], 1.5 mg/ml of proteinase K, and 15 mg/ml of lysozyme) was added to 100 mg of feces and incubated at room temperature for 10 min. After pretreatment, samples were treated with 1,200 μl of Qiagen RLT Plus buffer (from an AllPrep DNA/RNA minikit [Qiagen, Netherlands]) containing 1% (vol) beta-mercaptoethanol and were transferred into 2-ml sterile screw-cap tubes (Starstedt, Germany) filled with 1 ml of zirconia-silica beads (BioSpec Products, OK, USA) (<0.1 mm in diameter). Tubes were placed on a Vortex-Genie 2 mixer with a 13000-V1-24 Vortex adapter (Mo Bio, Inc., CA) and shaken at maximum speed for 15 min. Lysed fecal samples were homogenized using QIAshredder spin columns (Qiagen, Netherlands), and homogenized sample lysates were then extracted with an AllPrep DNA/RNA minikit (Qiagen, Netherlands) according to the manufacturer’s specifications. Extracted RNA and DNA were purified using Agencourt RNAClean XP and Agencourt AMPure XP (Beckman Coulter, Inc., CA) kits, respectively. Total RNA samples were subjected to rRNA depletion, and metatranscriptomic libraries were prepared using a ScriptSeq Complete Gold kit (epidemiology)-low input (Illumina, CA, USA). Metagenomic libraries were prepared with a Nextera XT DNA library preparation kit (Illumina, CA, USA). All libraries were sequenced on a HiSeq 2500 platform (Illumina, CA, USA) at an expected depth of 6 Gb/library.

### Sequencing data preprocessing.

The metagenomes and metatranscriptomes were preprocessed by removing low-quality reads (mean quality value of less than 25), trimming low-quality positions (quality less than 15), and removing reads less than 90 nucleotides in length using FastqMcf (74). Further quality control steps involved the removal of human reads and the reads from the Illumina spike-in (bacteriophage Phi-X174) by mapping the reads against the corresponding genomes with Bowtie 2 (75). Metatranscriptomes were additionally processed to remove rRNA by mapping the reads against 16S and 23S rRNA gene databases (SILVA_119.1_SSURef_Nr99_tax_silva and SILVA_119_LSURef_tax_silva [76]) and to remove contaminant adapters using trim_galore (http://www.bioinformatics.babraham.ac.uk/projects/trim_galore/) with the following parameters: -q 0, –nextera, and –stringency 5. The milk sample of mother-infant pair 4 at time point 1 was discarded from further analyses because of the low number of microbial reads (less than 400,000 bp) obtained after the quality control steps (see Table S1). All metagenomes and metatranscriptomes have been deposited in and are available at the NCBI Sequence Read Archive.

### Taxonomic and strain-level analysis.

Taxonomic profiling was performed with MetaPhlAn2 (77) (with default parameters) on the 23 metagenomic samples that passed the quality control. MetaPhlAn2 uses clade-specific markers for taxonomically profiling shotgun metagenomic data and to quantify the clades present in the microbiome with species-level resolution.

Strain-level profiling was performed with PanPhlAn (31) and a novel strain-level profiling method called StrainPhlAn (Truong et al., submitted). PanPhlAn is a pangenome-based approach that profiles the presence/absence pattern of species-specific genes in the metagenomes. The presence/absence profiles of the genes are then used to characterize the strain-specific gene repertoire of the members of the microbiome. PanPhlAn has been executed using the following parameters: –min_coverage 1, –left_max 1.70, and –right_min 0.30. PanPhlAn is available with supporting documentation at http://segatalab.cibio.unitn.it/tools/panphlan. StrainPhlAn is a complementary method based on analysis of SNVs that reconstructs the genomic sequence of species-specific markers. StrainPhlAn builds the strain-level phylogeny of microbial species by reconstructing the consensus marker sequences of the dominant strain for each detected species. The extracted consensus sequences are multiply aligned using MUSCLE version v3.8.1551 (78) (default parameters), and the phylogeny is reconstructed using RAxML version 8.1.15 (79) (parameters: -m GTRCAT and -p 1234). StrainPhlAn is available with supporting documentation at http://segatalab.cibio.unitn.it/tools/strainphlan.

### Functional profiling from metagenomes and metatranscriptomes.

The functional potential and transcriptomic analyses were performed with both HUMAnN2 (80) and PanPhlAn (31). HUMAnN2 selects the most representative species from a metagenome and then builds a custom database of pathways and genes that is used as a mapping reference for the coupled metatranscriptomic sample to quantify transcript abundances. We computed the normalized transcript abundance (NTA), which we define as the average coverage of a genomic region in the metatranscriptomic versus that in the corresponding metagenomic sample normalized by the total number of reads in each sample. PanPhlAn infers the expression of the strain-specific gene families by extracting them from the metagenome and matching them in the metatranscriptome. PanPhlAn has been executed using the following parameters: –rna_norm_percentile 90 and –rna_max_zeros 90.

### Profiling of DNA and RNA viruses.

We investigated the presence of viral and phage genomes by mapping the reads present in the metagenomes and metatranscriptomes against 7,194 viral genomes available in RefSeq (release 77). The average coverage and average sequencing depth were computed with SAMtools (81) and BEDTools (82).

The presence of the pepper mild mottle virus (PMMoV) was confirmed by mapping the reference genome (NC_003630) against the metatranscriptomic samples from the mother and infant of pair 4 and pair 5. In the mother and infant of pair 4, 424,510 and 119 reads were mapped, respectively, while in the mother and infant of pair 5, 1,444 and 61 of the reads were mapped, respectively. In the two mothers (pair 4 and pair 5), the values for breadth of coverage were 0.99 and 0.98 and for average coverage were 6,562 and 22, respectively. In the two infants (pair 4 and pair 5), the values for breadth of coverage were 0.6 and 0.5 and for average coverage were 1.81 and 0.95, respectively. Additionally, we extracted the shared fractions of the PMMoV genome present in both the mother and the infant of pair 4, together with the same regions of all the available reference genomes (*n* = 13 [specifically, accession no. LC082100.1, KJ631123.1, AB550911.1, AY859497.1, KU312319.1, KP345899.1, NC_003630.1, M81413.1, KR108207.1, KR108206.1, AB276030.1, AB254821.1, and LC082099.1]). The resulting sequences were aligned using MUSCLE version v3.8.1551 (default parameters), and the resulting alignment was used to build a phylogenetic tree with RAxML v. 8.1.15 (parameters: -m GTRCAT and -p 1234).

### Statistical analyses and data visualization.

The taxonomic and functional heat maps were generated using hclust2 (parameters: –f_dist_f Euclidean, –s_dist_f braycurtis, and –l) available at https://bitbucket.org/nsegata/hclust2. The multidimensional scaling plots were computed with the sklearn Python package (83).

Biomarker discovery (Fig. S7A) was performed by applying the linear discriminant analysis effect size (LEfSe) algorithm (84) (parameter: -l 3.0) on HUMAnN2 profiles. The two functional trees (Fig. S7A) have been automatically annotated with export2graphlan.py (GraPhlAn package) and displayed with GraPhlAn (85) using default parameters.

### Accession number(s).

All metagenomes and metatranscriptomes have been deposited and are available at the NCBI Sequence Read Archive under BioProject accession number PRJNA339914.
