# Supplementary figures and images for: Studying Vertical Microbiome Transmission from Mothers to Infants by Strain-Level Metagenomic Profiling
Source: mSystems. 2017 Jan 17;2(1):e00164-16. doi: 10.1128/mSystems.00164-16 (PMC5264247; doi:10.1128/mSystems.00164-16)

A

## Degradation

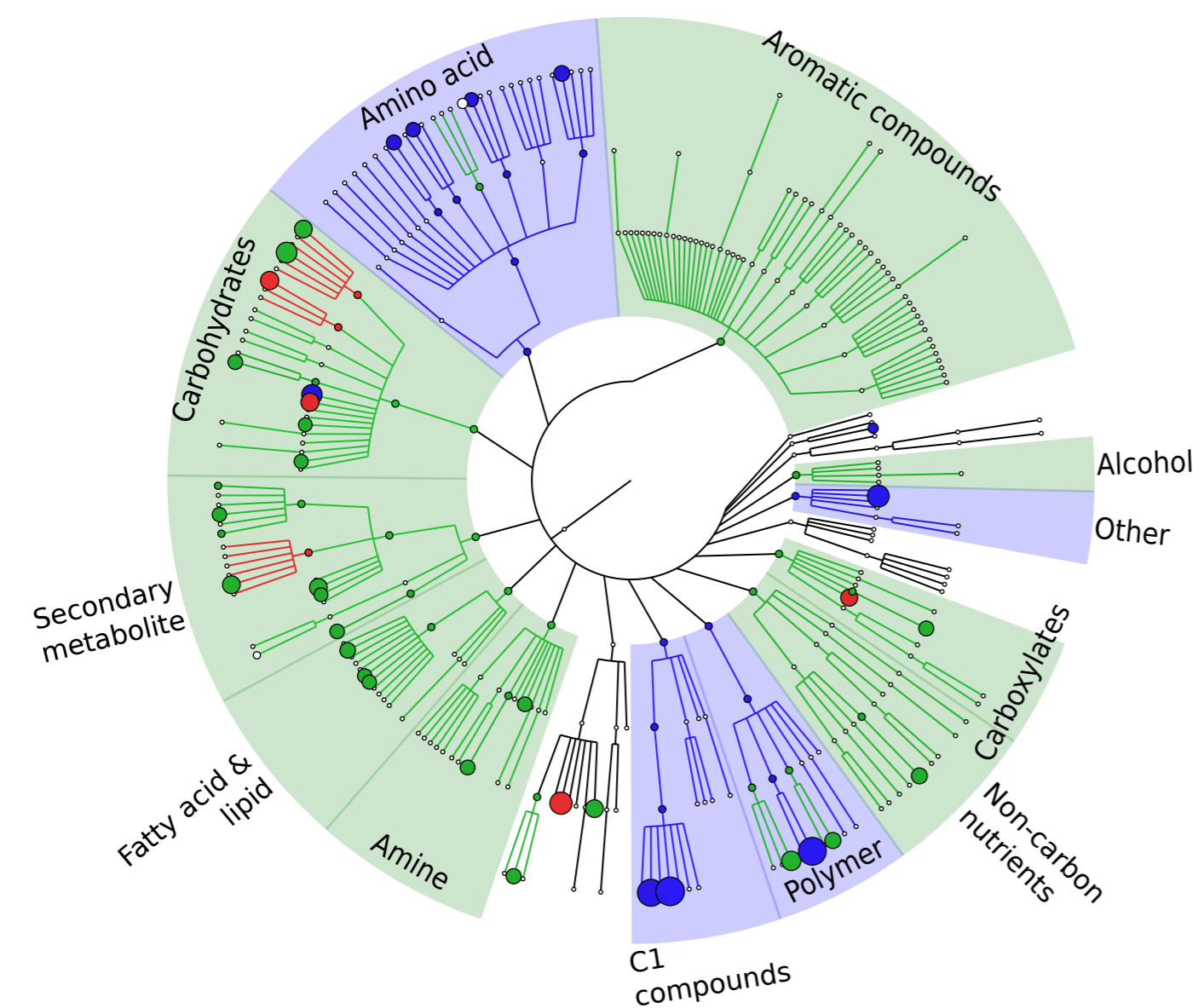

## Biosynthesis

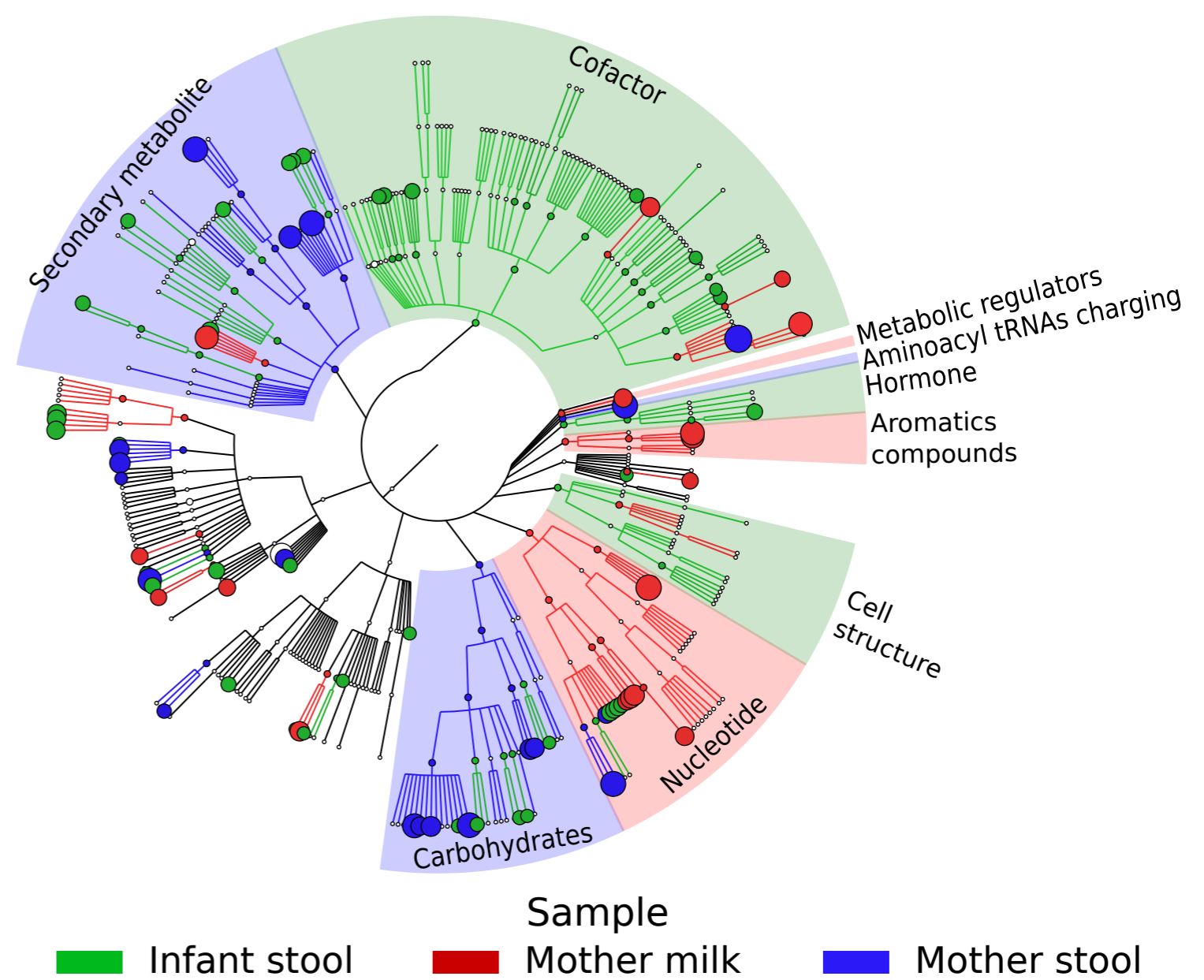

B

Mother stool Pair 5 T3

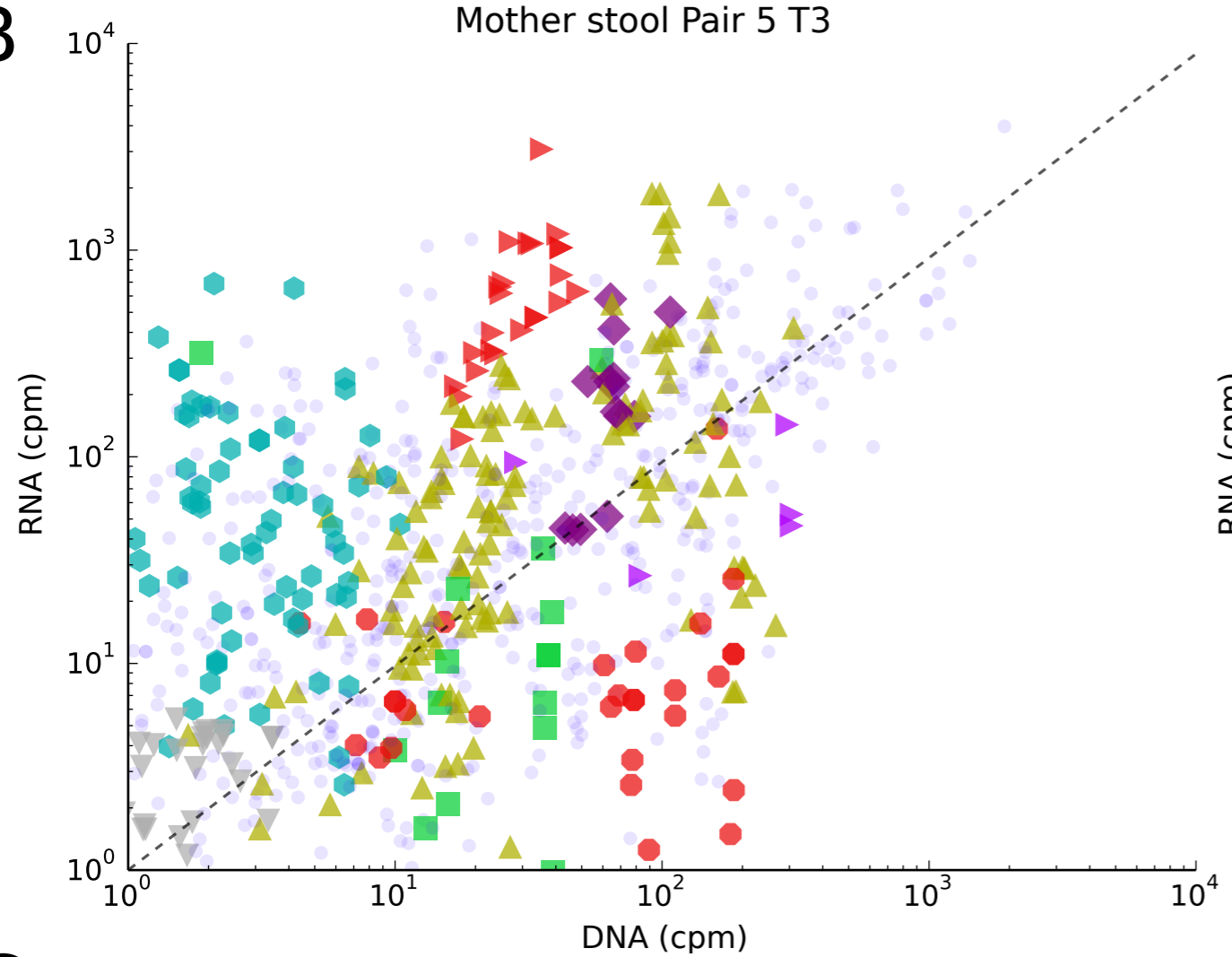

Infant stool Pair 5 T3

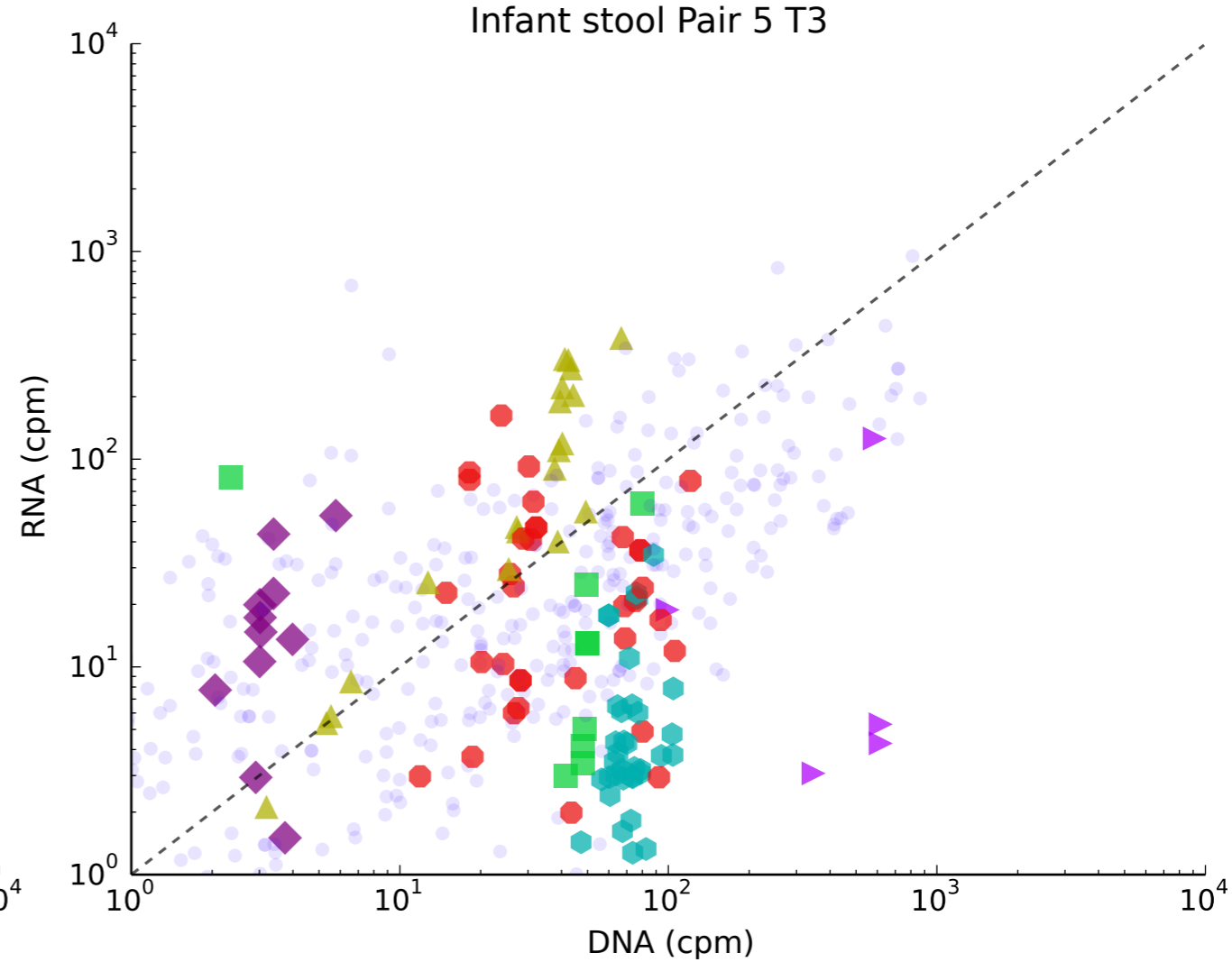

C

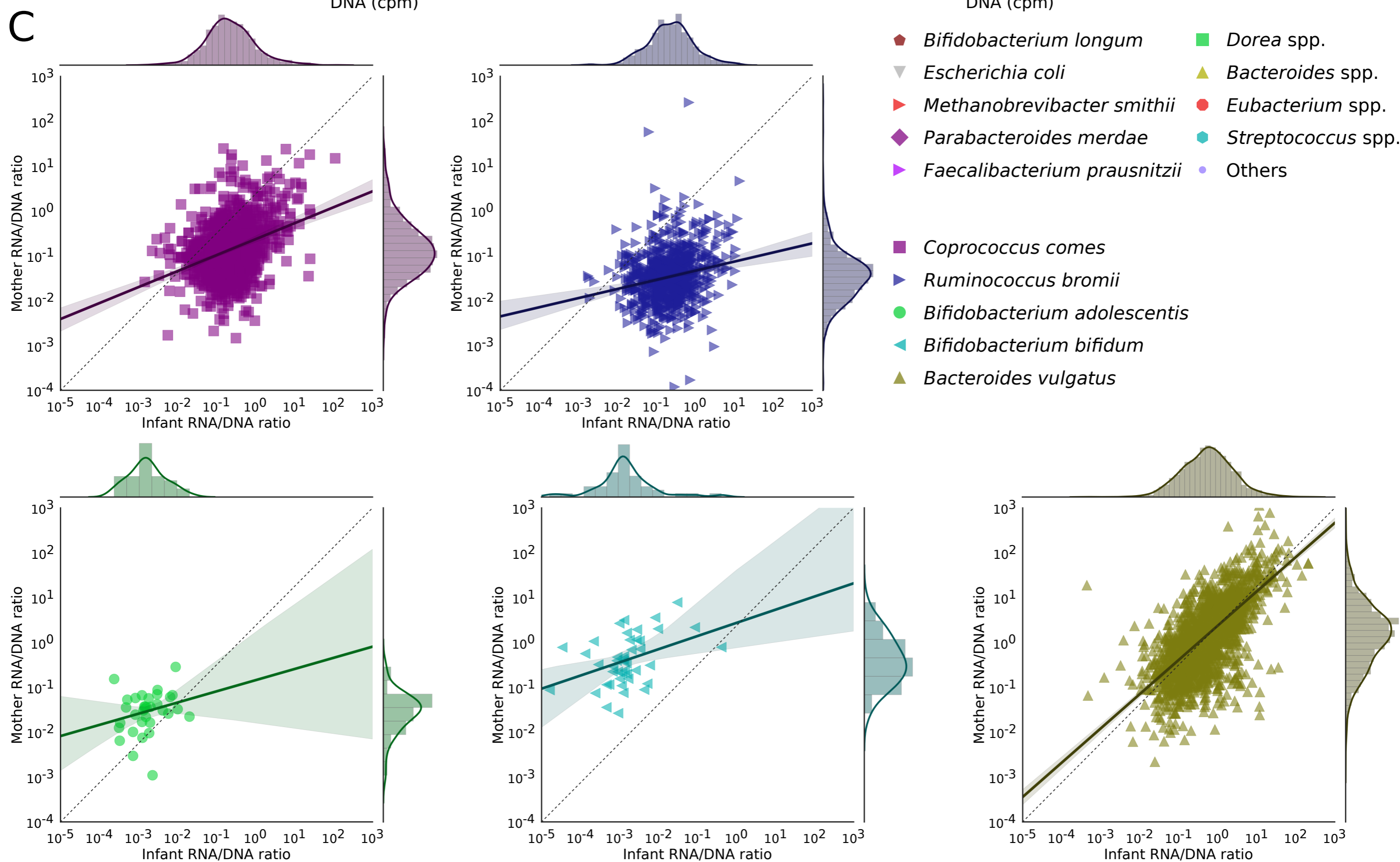

Supplement: FIG S7 [file sys001172080sf7.pdf]
